# Hepatorenal Index by B‐Mode Ratio Versus Imaging and Fatty Liver Index to Diagnose Steatosis in Alcohol‐Related and Nonalcoholic Fatty Liver Disease

**DOI:** 10.1002/jum.15991

**Published:** 2022-04-27

**Authors:** Maria Kjaergaard, Katrine Prier Lindvig, Camilla Dalby Hansen, Sönke Detlefsen, Aleksander Krag, Maja Thiele

**Affiliations:** ^1^ Department of Gastroenterology and Hepatology Odense University Hospital Odense Denmark; ^2^ Institute of Clinical Research, University of Southern Denmark Odense Denmark; ^3^ Department of Pathology Odense University Hospital Odense Denmark

**Keywords:** diagnostic accuracy, hepatorenal index, NASH, liver, steatosis

## Abstract

**Objectives:**

We aimed to evaluate the accuracy of the hepatorenal index by B‐mode ratio to diagnose hepatic steatosis, compared to ultrasound steatosis score, controlled attenuation parameter, and the fatty liver index using histology as the gold standard.

**Methods:**

We prospectively included participants with alcohol‐related or nonalcoholic fatty liver disease for same‐day noninvasive investigations and liver biopsy.

**Results:**

We included 137 participants, 72% male, median age 60 years (53–65) and body mass index 32 kg/m^2^ (28–38). Eighty percent had steatosis (S0/S1/S2/S3 = 20/37/24/19%). B‐mode ratio had moderate diagnostic accuracy for any steatosis (≥S1, area under the receiver operating characteristics curve [AUROC] = 0.79; 95% confidence interval 0.70–0.88), significant steatosis (≥S2, AUROC = 0.76; 0.66–0.85), and severe steatosis (=S3, AUROC = 0.74; 0.62–0.86), independent of disease etiology. The cutoff values to rule‐out and rule‐in any steatosis were 1.09 and 1.45. While B‐mode ratio and controlled attenuation parameter correlated poorly, their diagnostic accuracies were comparable to each other and to ultrasound steatosis scoring. Fatty liver index did not differ from B‐mode ratio in detecting any steatosis but had poor accuracy to detect higher steatosis grades. B‐mode ratio measurements failed in 12% of patients, compared to 1% for ultrasound steatosis scoring and 2% for controlled attenuation parameter.

**Conclusion:**

The hepatorenal index by B‐mode ratio diagnose steatosis with moderate accuracy in patients with alcohol‐related or nonalcoholic fatty liver disease, comparable to B‐mode ultrasound steatosis scoring and controlled attenuation parameter. However, its clinical use is limited by a high failure rate.

**Abbreviations**
ALD: alcohol‐related liver disease
AUROC: area under the receiver operating characteristics curve
BMI: body mass index
CAP: controlled attenuation parameter
CI: confidence interval
FLI: fatty liver index
HRI: hepatorenal index
IQR: interquartile range
MELD: model for end stage liver disease
NAFLD: nonalcoholic fatty liver disease
NPV: negative predictive value
PPV: positive predictive value
US: ultrasound

One in four of the global adult population has fatty liver disease and the prevalence is on the rise.^1^, ^2^, ^3^, ^4^ Simple hepatic steatosis can progress to steatohepatitis, fibrosis, and consequently cirrhosis, which is why we need reliable methods to diagnose and quantify steatosis.^5^

Liver biopsy is the reference method to diagnose and grade hepatic steatosis but is costly and of limited availability.^6^, ^7^ For this reason, ultrasound (US) is currently the first‐line diagnostic test.^7^ Evaluation of steatosis by US is an operator‐dependent, categorical assessment that can accurately detect moderate to severe steatosis but underestimates mild steatosis.^8^, ^9^, ^10^, ^11^ Several new US‐based techniques and blood‐based markers have been developed to improve diagnostic accuracy. Controlled attenuation parameter (CAP) detects hepatic steatosis with moderate accuracy but is inferior to US in detecting mild steatosis. Furthermore, CAP cannot quantify steatosis, and the cutoff values vary between etiologies.^12^, ^13^, ^14^ The blood‐based marker fatty liver index (FLI) is based on body mass index (BMI), waist circumference, gamma‐glutamyl transferase, and triglycerides, and has been proposed as a good marker to identify patients at risk of fatty liver disease.^15^

The hepatorenal index (HRI) is a promising US steatosis marker, which originally showed excellent accuracy for the diagnosis of any steatosis (≥5%).^16^, ^17^, ^18^ The HRI software calculates the ratio between the echogenicity of the hepatic parenchyma and the renal cortex on regular US B‐mode images (Figure 1). However, recent studies of HRI have reported diagnostic accuracies ranging from moderate to excellent, and with substantial differences in the proposed cutoff values.^19^, ^20^, ^21^, ^22^ Two studies investigated HRI using B‐mode ratio on the Aixplorer with good accuracy to detect any steatosis.^20^, ^22^ However, HRI has only been investigated in good quality images, with no evaluation of the failure rate, and never in alcohol‐related liver disease (ALD). Consequently, we aimed to evaluate the diagnostic accuracy of the HRI by B‐mode ratio (Aixplorer, Hologic) for steatosis assessment using liver biopsy as gold standard, and to compare B‐mode ratio with US steatosis scoring, CAP, and FLI, in a cohort of patients with ALD or nonalcoholic fatty liver disease (NAFLD), using liver biopsy as reference. This included defining rule‐in and rule‐out cutoff values for B‐mode ratio for mild (≥S1), significant (≥S2), and severe (=S3) steatosis, and subgroup analyses according to disease etiology. Secondary aims were to evaluate the failure rate of B‐mode ratio including predictors of failed measurements, and to explore quality criteria for optimal measurement of B‐mode ratio.

**Figure 1:**
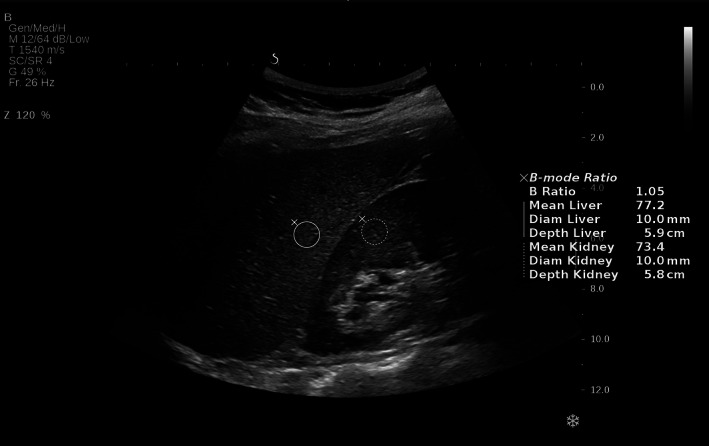
Hepatorenal B‐mode ratio in a normal liver using Aixplorer (Hologic).

## Materials and Methods

### Study Design

We performed a prospective, biopsy‐controlled, single center study in the region of Southern Denmark between 2018 and 2020 (ethics committee for Region of Southern Denmark approval S‐20170087, Danish Data Protection Agency 18/22692). This study is embedded in a larger screening study for fibrosis in fatty liver disease (ClinicalTrials.gov NCT03308916). All participants signed a consent form after written and oral information. The study adheres to the Declaration of Helsinki and the report follows the STARD guidelines for reporting of diagnostic tests.

### Participants

We included participants between 30 and 75 years, referred due to suspicion of liver fibrosis based on elevated liver stiffness measurements by transient elastography (≥8 kPa), and either ALD (prior or current alcohol overuse >25 g/day for women and >36 g/day for men for more than 5 years) or NAFLD (type 2 diabetes, metabolic syndrome, and/or BMI >30 kg/m^2^ with no alcohol overuse). We excluded participants if they had contraindications for liver biopsy, decompensated cirrhosis evidenced by US, known concurrent liver disease, severe alcoholic hepatitis, hepatic congestion, or bile duct dilatation evidenced by US, cancer, or other debilitating disease with an expected survival below 12 months, or lacking consent to a liver biopsy.

### Investigations

We performed all clinical investigations on the same day, after at least 6 hours of fasting. Anthropometric measurements included weight, height, waistline, and calculation of BMI. Laboratory tests were performed up to 3 days prior to the visit and included liver blood tests, and calculation of the model for end stage liver disease (MELD) score and FLI.^15^ Experienced nurse operators performed a new liver stiffness measurement by transient elastography and CAP with the FibroScan 502 touch (Echosens, France) according to standard.^13^ The operators were blinded to the histological steatosis grade, B‐mode ratio, US steatosis score, and FLI.

### US and B‐Mode Ratio

Three experienced US operators performed the US investigations using Aixplorer (Hologic) with a XC6‐1 convex probe. We evaluated the overall liver morphology, echo structure, and measured the skin to capsule distance. We defined US steatosis score as no steatosis (S0) if the liver was not hyperechoic; mild steatosis (S1) if there was increased hepatic echogenicity but no posterior attenuation; moderate steatosis (S2) if there was increased hepatic echogenicity with posterior attenuation evidenced as vessel blurring, but normal visualization of the diaphragm; and severe steatosis (S3) if there was increased hepatic echogenicity with severe attenuation resulting in vessel blurring and difficult visualization of the diaphragm.^23^

We measured the HRI by B‐mode ratio in the right intercostal space as part of the general US examination. The participants were lying in the supine position with their right arm above their head and normal breathing. We obtained five images that visualized both the right liver lobe and the right kidney in the same plane. On the frozen images, we placed the region of interests (ROIs) in the right liver parenchyma and the right kidney cortex. To get the best quality measurements we tried to position both ROIs at the same depth, avoiding artifacts, large ducts, vessels, masses, or cysts. The B‐mode ratio was directly calculated by the device. On each image, we placed two ROI at the same place: one with standard diameter of ROI (6 mm) and the other one with the largest possible diameter allowed by the US image to still retain optimal image quality to perform a B‐mode ratio measurement. If it was not possible to obtain B‐mode ratio measurements, they were classified as failure. After the study, one experienced operator (M.K.) evaluated all the scores and classified them as of either high or low quality according to whether the ROIs were at the same depth, and whether the images were free of artifacts, large ducts, vessels, masses, or cysts.

The US operators were blinded to the histological steatosis grade and FLI, but they semi quantitatively scored both US steatosis score and B‐mode ratio simultaneously.

### Liver Biopsy

We performed a percutaneous suction needle biopsy (17G Menghini needle; Hepafix; Germany) after the US examination. An experienced liver pathologist (S.D.) assigned steatosis grades as S0 (<5% hepatocytes with large fat vacuoles), S1 (5–33%), S2 (>33–66%), and S3 (>66% hepatocytes with large fat vacuoles).^24^ He also assigned fibrosis stage (0–4) according to the Pathology Committee of the NASH Clinical Research Network (NAS‐CRN).^24^ Lobular inflammation (0–3) and hepatocellular ballooning (0–2) were assessed according to the NAS‐CRN Activity Score.^24^ We considered the biopsies to be of sufficient quality when they were ≥10 mm in length and contained ≥6 portal tracts. The pathologist was blinded to the B‐mode ratio, US steatosis score, CAP, and FLI.

### Statistical Analyses

We reported quantitative continuous data as median and interquartile range (IQR) and categorical data as count and frequency. We tested between‐group differences with Wilcoxon rank sum test for continuous variables and Fisher's exact test for categorical variables. The histological steatosis grades were dichotomized into any steatosis (≥S1), significant steatosis (≥S2), and severe steatosis (=S3). We evaluated discriminative diagnostic accuracy using the area under the receiver operating characteristics curve (AUROC). Optimal cutoff was decided by the Youden Index. Rule‐out and rule‐in cutoff values were identified by maximizing the sensitivity and specificity above 90% respectively. The corresponding negative predictive value (NPV) and positive predictive value (PPV) were then determined. The DeLong test of equality of AUROC's were used to compare the diagnostic accuracy between the different methods as well as to test whether high‐quality measurements were better and if there was a difference among 2, 3, and 5 measurements. Spearman correlation analysis was performed to evaluate the correlation between the variables and histological steatosis grades, as well as for the correlation between B‐mode ratio and CAP. To evaluate predictors of failed measurements, we performed a multivariable regression analysis. We determined the sample size based on the ability to detect a specificity and a sensitivity of 90%, at an expected 72% prevalence of any steatosis.^14^ With confidence level set to 95% and 0.10 set to the maximally acceptable half width of the confidence interval (CI), we had to include 49 participants for the 90% sensitivity limit, and 124 for the 90% specificity limit.^25^ We considered *P* < .05 as statistically significant. We used STATA 16 (StataCorp, College Station, TX) for all statistical analyses.

## Results

### Participants

**Figure 2:**
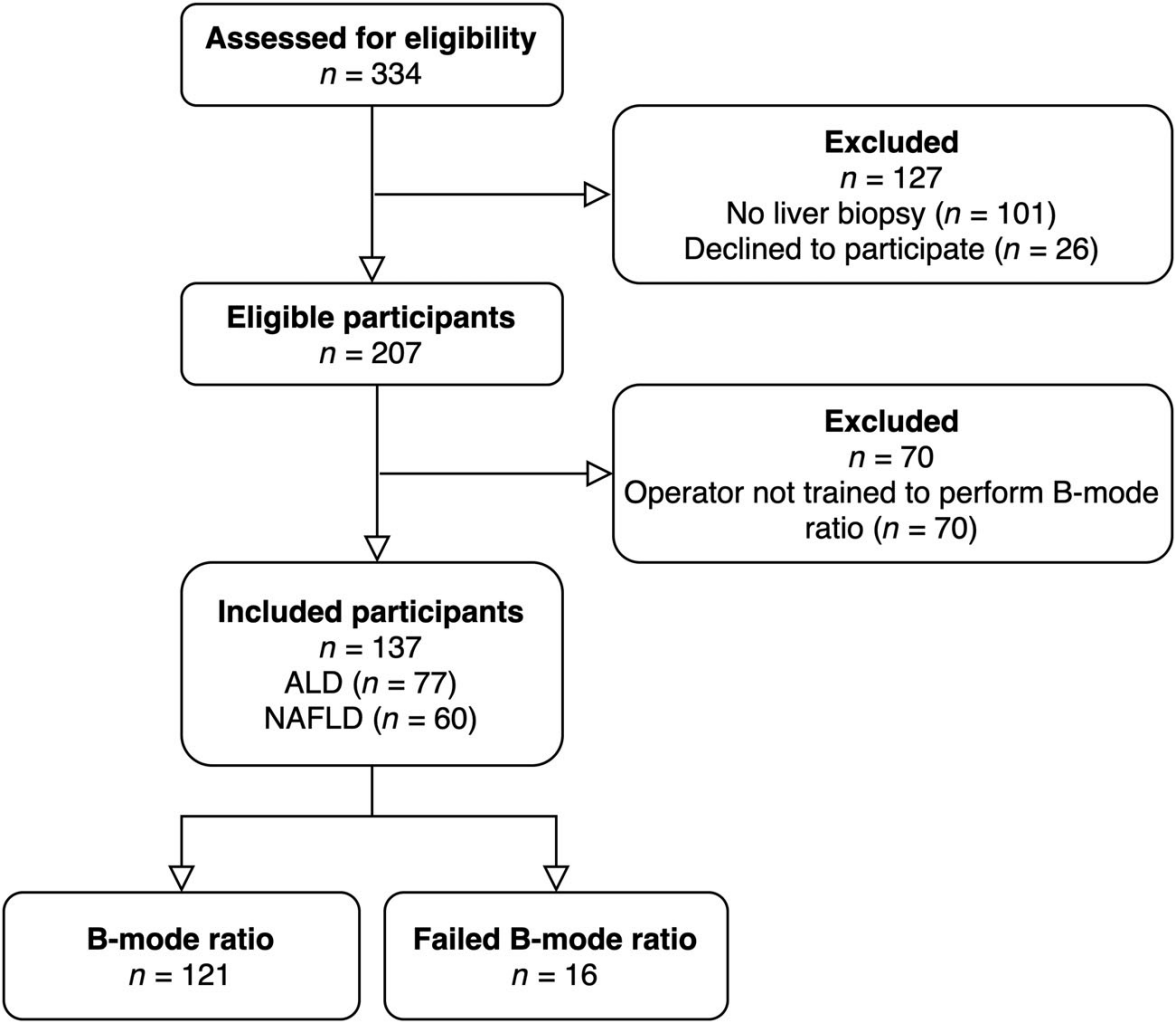
Study flow of the 137 included participants. It was technically possible to obtain a B‐mode ratio measurement in 121 participants, but B‐mode ratio failed in 16 (12%) of participants. ALD, alcohol‐related liver disease; NAFLD, nonalcoholic fatty liver disease.

From January 2018 to October 2020, we evaluated 334 participants for eligibility and included 137 participants in the study (Figure 2). In 121 (88%) of the included participants, it was possible to obtain a B‐mode ratio measurement. Ninety‐seven (72%) participants were males, the median age was 60 (53–65) years, and the median BMI was 32 (28–38) kg/m^2^ (Table 1). According to etiology, 77 (56%) had ALD and 60 (44%) had NAFLD. On histology, 109 (80%) of participants had steatosis. Besides postbiopsy pain, we did not register any adverse events from the biopsy procedures.

**Table 1:** Baseline Characteristics for the Total Population 137 Participants and in the Two Etiology Groups: ALD and NAFLD

	Total Population	ALD	NAFLD	*P*
	*n* = 137	*n* = 77	*n* = 60	
Age	60 (53–65)	61 (55–54)	58 (53–65)	.538
Gender, male (%)	98 (72)	66 (86)	32 (53)	.000
BMI (kg/m^2^)	32 (28–38)	30 (27–35)	35 (31–41)	.000
Waistline (cm)	110 (103–123)	109 (102–120)	114 (106–126)	.045
Skin to capsule distance (cm)	2.2 (1.9–2.7)	2.2 (1.8–2.5)	2.4 (2.1–2.9)	.005
Diabetes, yes	42 (31)	17 (22)	25 (42)	.016
Transient elastography	9.7 (7.5–15.8)	9.2 (7.8–14.4)	10.2 (7.7–16.1)	.646
Steatosis markers				
B‐mode ratio^a^	1.37 (1.15–1.60)	1.41 (1.15–1.65)	1.29 (1.17–1.56)	.165
Controlled attenuation parameter (CAP) (dB/m)^a^	337 (298–365)	334 (281–368)	346 (314–365)	.194
Ultrasonic steatosis score	18/28/39/51	13/20/20/24	5/8/19/27	.089
S0/1/2/3 (%)^a^	(13/20/28/37)	(17/26/26/31)	(8/13/32/45)	
Fatty liver index	92 (92–93)	92 (91–93)	93 (92–93)	.034
Histology				
Histological steatosis grade	28/50/33/26	19/25/18/15	9/25/15/11	.507
S0/1/2/3 (%)	(20/37/24/19)	(25/32/23/19)	(15/42/25/18)	
Kleiner fibrosis score	6/47/43/25/16	4/21/26/15/11	2/26/17/10/5	.383
F0/1/2/3/4 (%)	(4/34/31/18/12)	(5/27/34/19/14)	(3/43/28/17/8)	
Presence of steatohepatitis (%)	51 (37)	28 (36)	23 (38)	.860
Laboratory tests				
ALT (U/L)	41 (29–59)	39 (25–58)	41 (32–60)	.203
AST (U/L)	39 (27–57)	42 (26–58)	36 (27–49)	.458
Bilirubin (μmol/L)	9 (6–12)	9 (6–11)	9 (6–12)	.516
GGT (U/L)	85 (42–196)	102 (52–302)	58 (36–139)	.003
MELD (points)	7 (6–8)	7 (6–8)	7 (6–8)	.852

Continuous variables are reported as median (IQR) and categorical data as frequencies (%). The *P*‐value describes the difference between the two etiology groups (ALD versus NAFLD). The between‐group differences were calculated using Wilcoxon rank sum test for continuous variables and Fisher's exact test for categorical variables.

ALT, alanine aminotransferase; AST, aspartate aminotransferase; GGT, gamma‐glutamyl transferase.

^a^ It was not possible to evaluate B‐mode ratio in 16 (12%), ultrasonic steatosis score in 1 (1%), and CAP in 3 (2%).

### B‐Mode Ratio to Diagnose Steatosis

**Figure 3:**
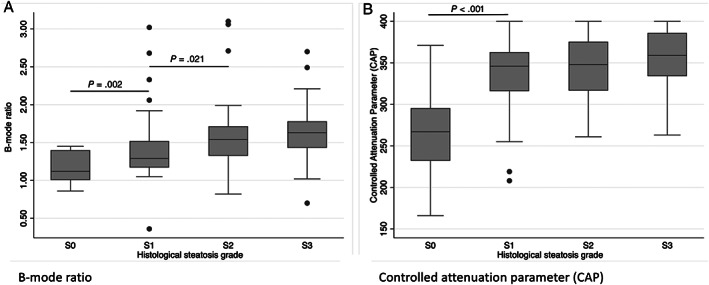
Distribution of **A**, B‐mode ratio and **B**, CAP according to histological steatosis grades (S0–S3). Significant *P*‐values are added in the graph.

**Figure 4:**
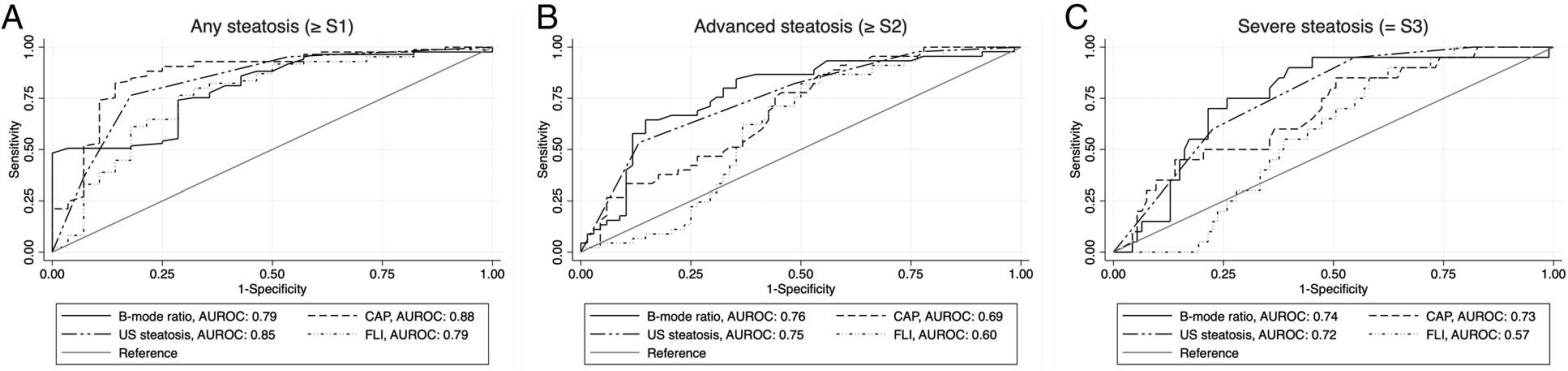
ROC curves for B‐mode ratio, CAP, US steatosis grades, and FLI to diagnose **A**, any steatosis (≥S1), **B**, significant steatosis (≥S2), and **C**, severe steatosis (=S3).

The median B‐mode ratio increased stepwise according to histological steatosis grades with a significant difference between the median B‐mode ratio of no histological steatosis (S0) and mild histological steatosis (S1) and between mild histological steatosis (S1) and moderate histological steatosis (S2) (Table 2 and Figure 3A). There was a moderate correlation between B‐mode ratio and histological steatosis grade (Rs = 0.506, *P* < .001). B‐mode ratio diagnosed any histological steatosis (≥S1), significant histological steatosis (≥S2), and severe histological steatosis (=S3) with moderate accuracy (Table 3 and Figure 4). Optimal cutoff values to rule‐out and rule‐in any histological steatosis (≥S1) were 1.09 and 1.45, respectively (Table 4). There was no significant difference in the distribution of B‐mode ratio or diagnostic accuracies between the ALD and NAFLD group (all *P* > .5).

**Table 2:** Distribution of B‐Mode Ratio, CAP, US Steatosis Score, and FLI According to Histological Steatosis Grades in a Cohort of 137 Patients With ALD and NAFLD

Histology	B‐Mode Ratio	CAP (dB/m)	US Steatosis Score (%)	FLI
S0	1.12 (1.01–1.40)	267 (232–196)	13/10/3/2 (46/36/11/7)	91 (90–92)
S1	1.29 (1.17–1.52)	346 (326–363)	4/9/23/13 (8/18/47/27)	93 (92–93)
S2	1.54 (1.33–1.72)	348 (317–376)	1/7/6/19 (3/21/18/58)	92 (92–93)
S3	1.63 (1.43–1.78)	359 (334–386)	0/2/7/17 (0/8/27/65)	93 (92–93)

B‐mode ratio, CAP, and FLI are reported as median (IQR) and US steatosis score as frequencies (%).

**Table 3:** Diagnostic Accuracy for B‐Mode Ratio, CAP, US Steatosis Grades, and FLI in a Cohort of 137 Patients With ALD and NAFLD

	B‐Mode Ratio	CAP		US Steatosis Score		FLI	
	AUROC (95% CI)	AUROC (95% CI)	*P*	AUROC (95% CI)	*P*	AUROC (95% CI)	*P*
≥S1	0.79 (0.70–0.88)	0.88 (0.81–0.96)	.174	0.85 (0.76–0.93)	.466	0.79 (0.68–0.89)	.648
≥S2	0.76 (0.66–0.85)	0.69 (0.60–0.78)	.304	0.75 (0.67–0.82)	.987	0.60 (0.50–0.70)	.021
=S3	0.74 (0.62–0.86)	0.73 (0.57–0.78)	.705	0.72 (0.63–0.81)	.934	0.57 (0.46–0.68)	.009

AUROC for diagnostic accuracy. *P*‐values for difference between the variables CAP, US steatosis score, and FLI, compared to B‐mode ratio, using the DeLong test for equality of ROC areas.

**Table 4:** Cutoff Values for B‐Mode Ratio to Rule‐In and Rule‐Out Steatosis in a Cohort of 137 Patients With ALD and NAFLD

	Cutoff Value	Sensitivity (%) (95% CI)	Specificity (%) (95% CI)	PPV (%) (95% CI)	NPV (%) (95% CI)	Correctly Classified (%)
≥S1						
Optimal	1.46	48 (38–59)	100 (88–100)	100 (92–100)	37 (26–49)	60
Rule‐out	1.09	91 (84–96)	43 (25–63)	85 (76–91)	60 (36–81)	80
Rule‐in	1.45	51 (40–61)	96 (82–100)	98 (89–100)	37 (26–49)	61
≥S2						
Optimal	1.48	64 (49–77)	85 (74–92)	74 (59–87)	77 (66–86)	76
Rule‐out	1.21	90 (78–97)	44 (32–56)	53 (42–64)	86 (71–95)	63
Rule‐in	1.67	38 (25–53)	90 (81–96)	73 (52–88)	67 (57–77)	69
=S3						
Optimal	1.39	86 (65–97)	60 (49–69)	32 (21–46)	95 (87–99)	64
Rule‐out	1.34	91 (71–99)	55 (44–65)	31 (20–43)	96 (88–100)	61
Rule‐in	1.92	18 (5–40)	91 (83–96)	31 (9–61)	83 (75–90)	78

Optimal cutoff by Youden Index. Rule‐out and rule‐in cutoff chosen based on sensitivity and specificity above 90% respectively.

### B‐Mode Ratio Compared to US Steatosis Score, Controlled Attenuation Parameter, and FLI

CAP increased significantly between no histological steatosis (S0) and mild histological steatosis (S1) (*P* < .001), but not between higher degrees of histological steatosis (Table 2 and Figure 3b). There was a poor correlation between CAP and B‐mode ratio (Rs = 0.277, *P* = .002). FLI was the same in all participants regardless of steatosis grade (Table 2). There was no difference between the diagnostic accuracies of B‐mode ratio, US steatosis score, and CAP (Table 3 and Figure 4).

### Failure Rate

B‐mode ratio had a high failure rate of 16 (12%) compared to US steatosis score (1 [1%]) and CAP (3 [2%]). One participant had a large part of the intestines between the right liver lobe and the peritoneum, which made it impossible to obtain either B‐mode ratio, US steatosis score, or CAP. All participants with a failed B‐mode ratio measurement had a liver biopsy with at least steatosis grade 1, their median BMI was 44 kg/m^2^, median waistline was 129 cm, and median skin to capsule distance was 3.2 cm. In multivariable regression analysis, skin to capsule distance independently predicted failed measurements with an odds ratio of 8.37 (95% CI 1.61–43.66, *P* = .012).

### Quality Criteria to Improve Diagnostic Accuracy

It was not possible to identify any quantitative criteria that improved the diagnostic accuracy of B‐mode ratio, neither when testing two, three, or five observations, nor when comparing fixed 6 mm versus largest possible ROI allowed by the US image to still retain optimal quality. By only using images of operator‐evaluated high quality, the diagnostic accuracy increased somewhat for any steatosis and significant steatosis (AUROC 0.83; 95% CI 0.73–0.92, and AUROC 0.85; 95% CI 0.76–0.94, respectively, *P* > .2 for comparison to all observations).

## Discussion

In this study, we found that the HRI by the B‐mode ratio (Aixplorer, Supersonic Imaging) diagnosed hepatic steatosis with moderate accuracy in patients with ALD and NAFLD, independent of etiology. While the diagnostic accuracy of B‐mode ratio was comparable to regular US steatosis score and CAP, it had a high failure rate, which limits its potential for clinical use.

The moderate diagnostic accuracy of B‐mode ratio in our study is lower than the initial studies on the HRI, that showed excellent diagnostic accuracy,^16^, ^17^, ^18^, ^20^ and is more in line with more recent prospective studies.^19^, ^21^, ^22^ Moret and colleagues recently investigated the HRI by B‐mode ratio with a slightly better diagnostic accuracy to diagnose any steatosis (≥S1), but similar results to diagnose higher degrees of steatosis.^22^

Unlike previous studies, we did not exclusively look at high‐quality images, which could contribute to the lower accuracy in our study. However, to relate the results to clinical practice, we believe that it is important to include all participants regardless of image quality. When we only looked at the operator‐evaluated high‐quality images, the diagnostic accuracy increased from moderate to good, however not significantly. This finding however emphasizes the importance of high‐quality measurements.

In this study, the etiology was equally distributed between patients with ALD and NAFLD, which are the most common causes of liver‐related morbidity and mortality in western countries. No previous studies have included a large proportion of participants at risk of ALD. We found no difference in the diagnostic accuracy of B‐mode ratio in the ALD and NAFLD cohort.

The cutoff values have varied between the different studies and most studies have used optimal cut off values instead of rule‐in and rule‐out cut off values. Our cutoff values to rule‐in and rule‐out any steatosis are in line with Moret and colleagues.^22^ B‐mode ratio seems to be a better tool to rule‐in steatosis than to rule‐out, but this is likely explained by a high prevalence of steatosis in the present, and other cohorts.

In this study, we show that regular US steatosis scoring and CAP detect any steatosis (≥S1) with good accuracy. The easy access of US and its wide implementation among clinicians is an advantage of regular US steatosis scoring. Our results indicate that regular US steatosis scoring is effective to detect steatosis in a cohort with a high prevalence of steatosis. However, if a FibroScan equipment is available, CAP is a good alternative to B‐mode ratio or regular US steatosis score. In contrast, we did not find evidence in favor of FLI for detecting steatosis. However, in unselected cohorts, FLI may be used to decide which patients need an US or CAP measurement. Based on this study, it is not possible to predict how any of these techniques will perform in a low prevalent population, for instance as a screening tool among an unselected population in primary care.

We are the first to evaluate the failure rate of HRI. Previous studies have excluded participants that did not have high quality measurements. We found a high failure rate compared with US steatosis scoring and CAP, even among trained personnel. This high failure rate limits the clinical use of B‐mode ratio. In particular, the skin‐capsule distance predicted measurement failure. Consequently, higher BMI and abdominal obesity in patients suspected of fatty liver disease will likely lead to even higher failure rates. In a leaner population, with a lower prevalence of steatosis, the B‐mode ratio may show higher accuracy and improved applicability. As such, we speculate that B‐mode ratio may be a useful supplement to regular US steatosis scores, for example in case of operator disagreement, or when a continuous measure is needed, for instance to monitor treatment efficacy.

Our results are strengthened by the study being prospective, biopsy‐controlled, with a well‐defined and clinically relevant cohort. All investigations were performed on the same day, which is a major strength since liver steatosis varies fast. Another strength of this study is that the US operators were blinded to the histological steatosis grades and the pathologist was blinded to the B‐mode ratio, US steatosis score, and CAP. The limitations of this study were that we did not evaluate intra‐ and interobserver variance. The same operator performed both B‐mode ratio and US steatosis score and was not blinded to CAP, limiting the comparison analyses. The study cohort was selected based on risk factors for fatty liver disease and suspected of having advanced fibrosis, resulting in a high prevalence of steatosis. Other semiquantitative US steatosis scores are available and may be more accurate than regular US steatosis score.^26^, ^27^ However, they are not validated in ALD, which is why we used CAP as comparator. Since B‐mode ratio does not outperform regular US steatosis score, it is not likely that B‐mode ratio would have outperformed other steatosis scores based on US imaging.

Since B‐mode ratio is on a continuous scale, it may have a potential role as a monitoring tool to evaluate treatment efficacy. Further studies are needed to evaluate the potential of B‐mode ratio to monitor steatosis.

## Conclusion

The HRI by B‐mode ratio has a moderate accuracy for the noninvasive diagnosis of hepatic steatosis in patients with ALD or NAFLD, comparable to regular US steatosis scoring or CAP. However, B‐mode ratio has a higher failure rate, which limits its clinical use.
